# Not Just for Workers: Maternal Exposure to Ambient Benzene Linked to Spina Bifida in Infants

**DOI:** 10.1289/ehp.119-a133a

**Published:** 2011-03

**Authors:** Julia R. Barrett

**Affiliations:** **Julia R. Barrett**, MS, ELS, a Madison, WI–based science writer and editor, has written for *EHP* since 1996. She is a member of the National Association of Science Writers and the Board of Editors in the Life Sciences

Occupational exposure to hazardous air pollutants such as benzene has been linked in some studies to neural tube defects (NTDs), yet to date no one had studied whether exposure to ambient levels of benzene may similarly lead to adverse outcomes. A new study now reveals a positive association between exposure to ambient benzene in outdoor air and increased prevalence of spina bifida **[*EHP*** **119(3):397–402; Lupo et al.].**

NTDs are a common type of birth defect. They arise when the neural tube fails to close during fetal development, leading to spina bifida (incomplete spinal column formation) or anencephaly (incomplete brain and skull formation). Both genetic and environmental factors, particularly inadequate folic acid intake, appear to play a role in NTDs.

The Texas Birth Defects Registry provided data from 1 January 1999 to 31 December 2004 on 1,108 newborn infants, stillborn infants, and electively terminated fetuses with NTDs. A random set of 4,132 unaffected infants born during the same period served as a control group. Ambient air levels of benzene, toluene, ethylbenzene, and xylene were estimated at the census-tract level using the U.S. Environmental Protection Agency’s 1999 Assessment System for Population Exposure Nationwide (ASPEN) paired with mothers’ residential addresses at the time they gave birth. After exclusions for missing data and known chromosomal abnormalities or syndromes, 533 spina bifida cases, 303 anencephaly cases, and 3,695 control cases remained for analysis.

Mothers with the highest estimated benzene exposure (≥3 μg/m3 in ambient air) were 2.3 times as likely as mothers in the reference group to bear children with spina bifida. The relationship between benzene exposure and spina bifida was statistically significant for most levels of exposure above the reference value, but the dose–response relationship was not monotonic (that is, the odds of risk did not increase consistently with each increase in exposure level). Associations between other solvents and spina bifida and between individual solvents and anencephaly also were observed but were not statistically significant.

The study has several potential limitations including possible exposure misclassification, the availability of pollutant data for only 1 year of the study period, and limited information on potential confounders, such as maternal folic acid intake. However, these limitations are at least partially offset by ASPEN’s high-quality exposure estimates, the likelihood that pollutant levels were stable during the study years, and mandatory folic acid fortification of foods.

This study is the first to suggest spina bifida prevalence is associated with maternal exposure to ambient air benzene levels. Further study of exposure, genotypes, and maternal nutrient status are needed to confirm this finding.

